# LncRNA MIAT can regulate the proliferation, apoptosis, and osteogenic differentiation of bone marrow-derived mesenchymal stem cells by targeting miR-150-5p

**DOI:** 10.1080/21655979.2021.2011632

**Published:** 2022-03-12

**Authors:** Fei Wang, Huimin Deng, Jimin Chen, Zhaobin Wang, Ruofeng Yin

**Affiliations:** a Department of Orthopedics, China-Japan Union Hospital, Changchun, China; b Jilin Medical Products Administration, Changchun, China; c Department of Geriatrics, Yueyang Hospital of Integrated Traditional Chinese and Western Medicine, Shanghai University of Traditional Chinese Medicine, Shanghai, China; d Department of Orthopedics, Liaohe Hospital, Liaoyuan, China

**Keywords:** LncRNA miat, osteoporosis, miR-150-5p, bmscs

## Abstract

Osteoporosis (OP) is a systemic bone metabolic disease with complicated pathogenesis and is difficult to cure clinically. The regulatory mechanisms of OP are needed to be further investigated. In the present study, we focused on the role of myocardial infarction-associated transcript (MIAT) in OP development and examined the underlying mechanism. The serum expression levels of MIAT in samples from patients with OP and healthy controls were compared using quantitative reverse transcription-PCR (qRT-PCR). The dual-luciferase reporter assay was used to confirm the relationship between MIAT and its potential target microRNA, i.e., miR-150-5p. Moreover, bone marrow-derived mesenchymal stem cells (BMSCs) were cultured and transfected with MIAT shRNA, with or without miR-150-5p inhibitor. EdU staining and colony formation analysis were performed to determine the proliferation ability of these cells. Furthermore, the TUNEL assay and flow cytometry were used to assess BMSC apoptosis. Finally, RT-PCR and Western blot assays were employed to assess the expression of osteogenic differentiation biomarkers. Compared with controls, the expression of MIAT was significantly increased, whereas that of miR-150-5p was markedly decreased in patients with OP. MIAT and miR-150-5p expression levels exhibited a strong negative correlation. Furthermore, osteogenic differentiation indicators were suppressed in serum of OP patients. MIAT was downregulated, and miR-150-5p was upregulated in induced to osteogenic differentiation BMSCs. Furthermore, downregulation of MIAT dramatically promoted osteogenic differentiation, increased proliferation, and inhibited apoptosis in BMSCs; miR-150-5p inhibitor abrogated the effects of MIAT. In conclusion, lncRNA MIAT can regulate the proliferation, apoptosis, and osteogenic differentiation of BMSCs.

## 1. Introduction

As the most prevalent progressive bone disease, osteoporosis (OP) dramatically limits patient activity and greatly increases the medical burden [1]. OP is characterized by low bone mass, potentially resulting in bone rarefaction and fractures [2]. It is well known that bone is a continuously and dynamically modified tissue, predominantly consisting of osteoblasts and osteoclasts. Osteoblasts promote bone formation, whereas osteoclasts can induce bone resorption [3]. The imbalance between osteoblasts and osteoclasts has been indicated as the primary factor resulting in OP and other bone metabolic processes [4]. Bone marrow-derived mesenchymal stem cells (BMSCs), a known source of osteoblasts, maintain a balance between bone formation and bone resorption [5,6]. Therefore, an in-depth investigation into the roles of BMSCs in OP is critical for early diagnosis, as well as disease management [7].

Myocardial infarction-associated transcript (MIAT), a long non-coding RNA (lncRNA), reportedly plays a key role in the development of myocardial infarction [8]. Recent studies have shown that MIAT is abnormally expressed in microvascular dysfunction [9], paranoid schizophrenia [10], and cancer [11]. In contrast, MIAT was found to be downregulated in adipose tissue-derived stem cells (ASCs) undergoing osteogenic differentiation [12]. For instance, MIAT knockdown obviously ameliorated diabetes mellitus-induced retinal microvascular dysfunction by regulating miR-150-5p [9]. MIAT contributed to non-small cell lung cancer proliferation and metastasis through MMP9 activation [11]. Moreover, MIAT silencing can induce the osteogenic process of mesenchymal stem cells (MSCs) [13,14]. Nevertheless, the role of MIAT in OP warrants further investigation.

LncRNAs are reportedly involved in exerting biological functions [15]. Osteoblast differentiation and osteogenesis are regulated by different miRNAs [16]. MIAT targets distinct miRNAs in different diseases. For instance, MIAT, which time-dependently increases during BMSC differentiation, induces BMSC differentiation into endothelial cells by targeting miR-200a [17]. Moreover, MIAT sponges miR-150-5p to regulate oxidative stress and modulate the function of human lens epithelial cells [18]. In contrast, miR-150 induces osteoblast differentiation [19] and inhibits osteoclast growth [20].

Accordingly, in the present study, we focused on the roles of MIAT and miR-150-5p in OP. Interaction between MIAT and miR-150-5p, and their effects on osteoblast differentiation of BMSCs were investigated, which may provide a novel treatment idea for OP.

## 2. Materials and Methods

### 2.1 Patients

Herein, we enrolled 30 healthy volunteers and 30 patients with OP, and took blood samples and isolated serum samples. Patients with OP presenting with hip fractures requiring surgical intervention, a history of cardiovascular diseases, cancers, diabetes, or other metabolic problems were excluded. Each participant provided informed consent before study initiation. The study protocol was approved by the Ethics Committee of the Liaohe Hospital.

### 2.2 Cell culture

HEK 293 T cells (ATCC, Manassas, VA) were cultured using Dulbecco’s Modified Eagle Medium (DMEM; Thermo Fisher Scientific, Inc., Waltham, MA, USA), supplemented with 10% fetal bovine serum (FBS; Gibco; Thermo Fisher Scientific, Inc.) and 1% penicillin/streptomycin (Invitrogen) [21]. The cells were maintained at 95% humidity and 5% CO_2_ at 37°C.

### 2.3 Isolation and culture of BMSCs

Gradient centrifugation was performed to isolate MSCs from the bone marrow of rats. In brief, four male Sprague-Dawley rats were housed under standard conditions and allowed to access food and water *ad libitum*. Bone marrow aspirates were placed in MEM medium (Gibco), supplemented with 10% FBS for 24 h at 95% humidity and 5% CO_2_ at 37°C [22]. Suspended cells were removed, and then cells were cultured for approximately 10 days. On reaching ~75% confluency, cells were harvested for subsequent experiments.

## 2.4 shRNA transfection

MIAT (shMIAT-1 5ʹ-GGTCAGGATTAGTGGTCATTC-3ʹ and shMIAT-2 5ʹ-GGTGATTACCGTGCACCTTGA-3ʹ) and the negative control (NC; TTCTCCGAACGTGTCACGTTTC) were purchased from GenePharma Co. (Suzhou, China). shRNA transfection was performed using Lipofectamine 2000 (Invitrogen) [23].

### 2.5 Dual-luciferase reporter assay

The binding sites between miR-150 and MIAT were predicted using StarBase 3.0. To verify that MIAT targeted miR-150, HEK 293 T cells (2 × 10^4^ cells/mL, 200 μL) were plated into 48-well plates. Then, cells were treated with 400 ng wild-type (WT) MIAT or mutant (MUT) MIAT 3ʹ-UTR. After incubation for 6 h, HEK 293 T cells were cultured in DMEM for an additional 48 h. Luciferase activity was determined using a commercial kit (Promega Corporation) [24].

## 2.6 5-Ethynyl-2ʹ-deoxyuridine staining (EdU)

EdU was used to examine BMSC proliferation [25]. Briefly, BMSCs were incubated with EdU (50 μM) for 2 h. Then, BMSCs were obtained and stained using a kit (Ribobio). Finally, images were visualized and photographed using a microscope (Olympus).

### 2.7 Colony formation assay

In brief, BMSCs were seeded into 6-well plates and cultured for 7 days, with the culture medium replaced every 2 days. Thereafter, BMSCs were stained with crystal violet (0.1%) for 10 min [26]. Colonies were visualized and counted using a microscope (Nikon, Tokyo, Japan).

### 2.8 Alkaline phosphatase (ALP) assessment

The cells were fixed with 4% paraformaldehyde (PFA) for 10 min at room temperature. Then, cells were seeded and stained with ALP for 20 min [27]. The images were captured using a scanner. An ALP Assay Kit was used to detect ALP activity in accordance with the manufacturer’s protocol.

### 2.9 Cell apoptosis

Cells were stained with the propidium iodide (PI)/Annexin V-FITC kit (Sigma) in the dark for 10 min at room temperature [28]. Then, the cells were counted by flow cytometry (BD Biosciences).

### 2.10 Terminal-deoxynucleotidyl Transferase Mediated Nick End Labeling (TUNEL) staining

TUNEL staining was used to assess BMSC death [28]. Briefly, BMSCs were fixed and treated with a TUNEL staining kit (Beyotime). Then, images from six randomly selected fields were captured using a microscope (Olympus).

### 2.11 Reverse transcription-polymerase chain reaction (qRT-PCR)

RNA samples were extracted from collected serum and BMSCs using a commercially available kit (Takara, Japan). cDNA was synthesized, and PCR was performed using a Real-Time PCR Detection System (Bio-Rad, USA). The relative levels of MIAT, ALP, osteocalcin (OCN), and RUNX2 were normalized to GAPDH. miR-150-5p expression was standardized to U6. The sequences of the primers were as follows: Human MIAT forward: 5ʹ-CATGGCCTCCGTAGTAACTCAC-3ʹ, reverse: 5ʹ-TCAAACCCCAGCCACTCTTC-3ʹ; Human GAPDH forward:5ʹ- CATCATCCCTGCCTCTACTGG-3ʹ, reverse 5ʹ- GTGGGTGTCGCTGTTGAAGTC-3ʹ; Rat OCN forward: 5ʹ- CCAGCGACTCTGAGTCTGACAA-3ʹ, reverse: 5ʹ-AACGGTGGTGCCATAGATGC-3ʹ; Rat ALP forward: 5ʹ-TGGACCTCATCAGCATTTGG-3ʹ, reverse: 5ʹ- GAGGGAAGGGTCAGTCAGGTT-3ʹ; Rat RUNX2 forward: 5ʹ-TACTCTGCCGAGCTACGAAATG-3ʹ, reverse: 5ʹ-TGAAACTCTTGCCTCGTCCG-3ʹ; Rat MIAT forward: 5ʹ-GCGAGCAATCTGAAGATCCTG-3ʹ, reverse: 5ʹ-GCTCTACCCCATCTCCAGAGAC-3ʹ; Rat GAPDH forward: 5ʹ-CGCTAACATCAAATGGGGTG-3ʹ, reverse: 5ʹ-TTGCTGACAATCTTGAGGGAG-3ʹ; hsa-miR-150-5p forward: 5ʹ- TCCCAACCCTTGTACCAGTG-3ʹ, reverse 5ʹ- CTCAACTGGTGTCGTGGAGTC-3ʹ; Human U6 forward: 5ʹ- CTCGCTTCGGCAGCACAT-3ʹ, reverse: 5ʹ- AACGCTTCACGAATTTGCGT-3ʹ; rno-miR-150-5p forward: 5ʹ- TCCCAACCCTTGTACCAGTG-3ʹ, reverse: 5ʹ- CTCAACTGGTGTCGTGGAGTC-3ʹ; Rat U6 forward: 5ʹ- CCTGCTTCGGCAGCACAT-3ʹ, reverse: 5ʹ- AACGCTTCACGAATTTGCGT-3ʹ.

### 2.12 Western blotting

Protein extracts (15 μg/lane) were subjected to 10% sodium dodecyl sulfate gel electrophoresis. The protein extracts were then transferred to a polyvinylidene fluoride membrane (Millipore) and incubated with primary antibodies at 4°C overnight. On day 2, the membrane was incubated with secondary antibodies for 2 h at room temperature. Protein bands were visualized using an ECL Western blot Kit (CWBIO). Each protein level was normalized to that of GAPDH.

### 2.13 Statistical analysis

Statistical analyses were performed using GraphPad Prism 7.0 (GraphPad Software, La Jolla, CA, USA). Data are expressed as mean ± standard deviation (SD). Comparison of two groups was performed using Student’s t-tests. Comparison of more than two groups was performed using analysis of variance. Statistical significance was set at P < 0.05.

## 3. Results

### 3.1 MIAT and miR-50-5p were aberrant expressed in patients with OP

**Figure 1.**
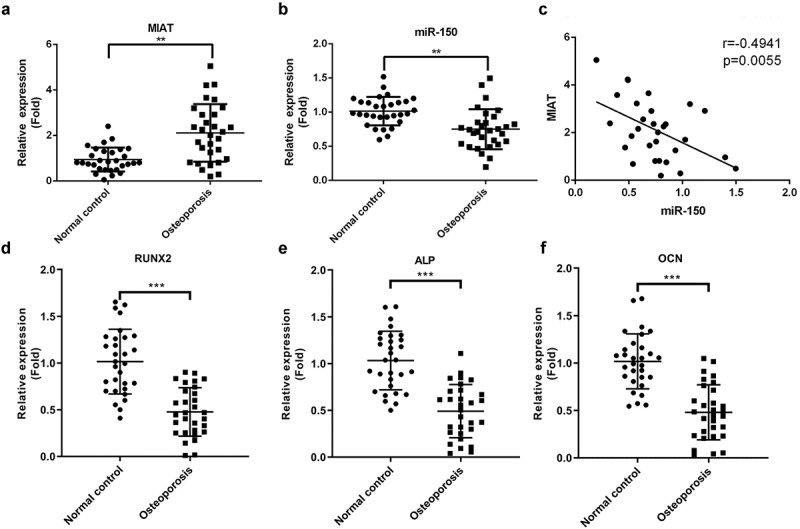
**Expression level of MIAT and miR-150-5p in serum samples of OP patients and healthy controls**. MIAT was increased (a) and miR-150-5p was decreased (b) in the serum from patients with postmenopausal OP compared with healthy volunteers. A significant negative correlation was found between the levels of MIAT and miR-150-5p in serum of the OP patients (c). Osteogenic differentiation indicators RUNX2, ALP and OCN were evaluated in the serum from patients with postmenopausal OP compared with healthy volunteers (d-f). **p < 0.01, ***p < 0.001.

Based on the qRT-PCR analysis, we observed that MIAT expression was increased (Figure 1a, *p* < 0.01), while miR-150-5p was decreased (Figure 1b, *p* < 0.01) in serum samples from patients with OP. Additionally, correlation analysis revealed that MIAT and miR-150-5p expression levels were negatively correlated (Figure 1c, r = - −0.4941, *p* = 0.0055). Furthermore, osteogenic differentiation-related proteins including Runx2, ALP, and OCN were dramatically lower in serum of patients with OP than those in healthy control group (Figure 1d-F, *p* < 0.001).

### 3.2 MIAT targets miR-150-5p

**Figure 2.**
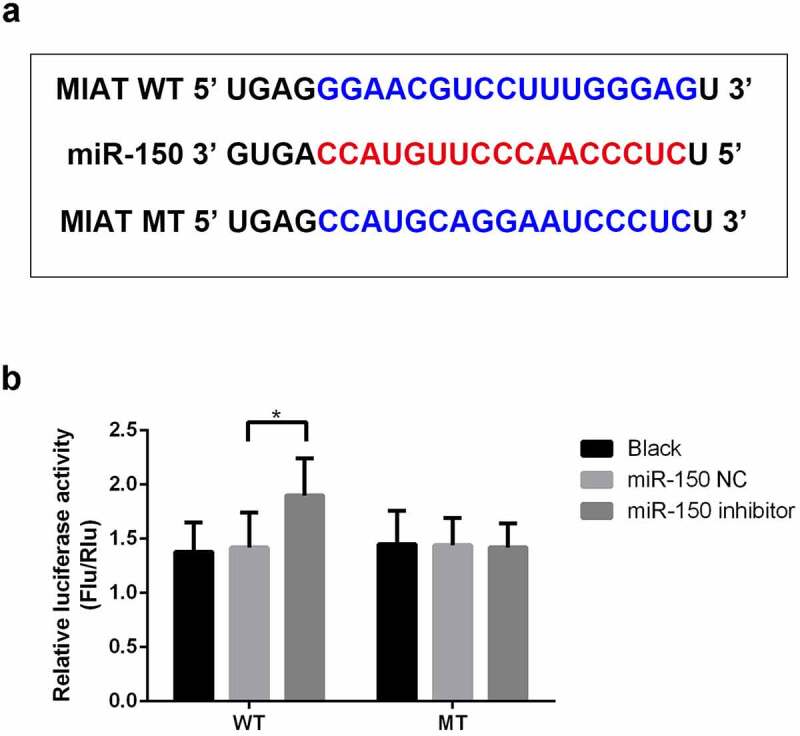
**miR-150-5p is a target of MIAT**. MIAT was predicted to target miR-150-5p (a). Compared with miR-NC inhibitor group, miR-150-5p inhibitor significantly increased the luciferase activity of the WT-MIAT (b). *p < 0.05.

Using bioinformatics tools, MIAT was predicted to target miR-150-5p (Figure 2a). As shown in Figure 2b, luciferase reporter analysis revealed that miR-150-5p inhibitor markedly upregulated luciferase activity in the WT-MIAT group while demonstrating no significant effect in the MUT-MIAT group (Figure 2b, *p* < 0.05).

### 3.3 MIAT is downregulated during osteogenic differentiation of BMSCs

**Figure 3.**
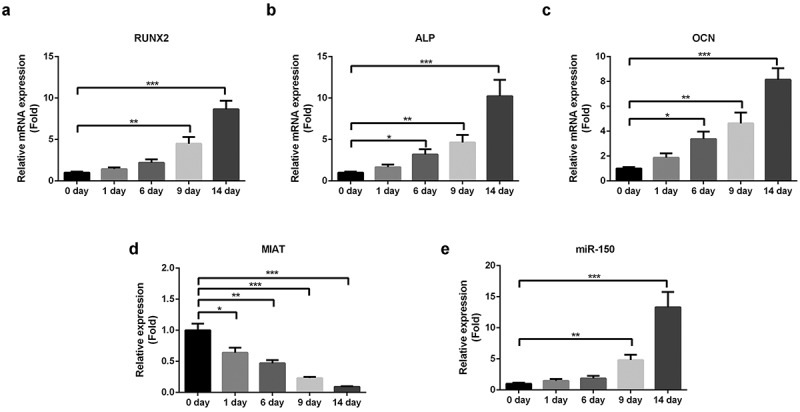
**MIAT is significantly downregulated during the osteogenic differentiation of BMSCs**. Osteogenic differentiation of BMSCs was evidenced by the increased expression of RUNX2, ALP and OCN (a-c). MIAT was decreased and miR-150-5p was increased time-dependently during the osteogenic differentiation of BMSCs (c and d). *p < 0.05, **p < 0.01, ***p < 0.001.

The expression level of MIAT in BMSCs was determined by qRT-PCR. As shown in Figure 3, osteogenic differentiation of BMSCs was confirmed by increased levels of osteogenic markers (Figure 3a-c). On the contrary, the expression of MIAT was decreased (Figure 3d), while miR-150-5p was increased during osteogenic differentiation in a time-dependent manner (Figure 3e).

### 3.4 Transfection of shMIAT and miR-150-5p inhibitor in BMSCs

**Figure 4.**
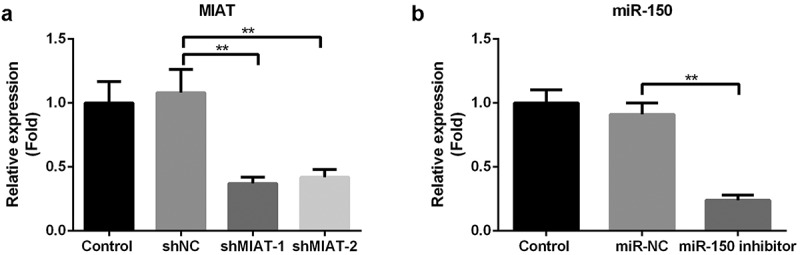
**Transfection of shMIAT and miR-150-5p inhibitor in BMSCs**. The level of MIAT was significantly inhibited by the transfection of shMIAT-1 and shMIAT-2 in BMSCs (a). miR-150-5p inhibitor significantly decreased the level of miR-150-5p in BMSCs (b). **p < 0.01.

Compared with the shNC group, shMIAT-1 and shMIAT-2 markedly decreased the expression level of MIAT in BMSCs, especially in shMIAT-1 treated cells (Figure 4a, *p* < 0.01). Therefore, shMIAT-1 was used in the present study. In contrast, the miR-150-5p inhibitor dramatically downregulated miR-150-5p expression in BMSCs (Figure 4b, *p* < 0.01).

### 3.5 MIAT promotes the proliferation of BMSCs by targeting miR-150-5p

**Figure 5.**
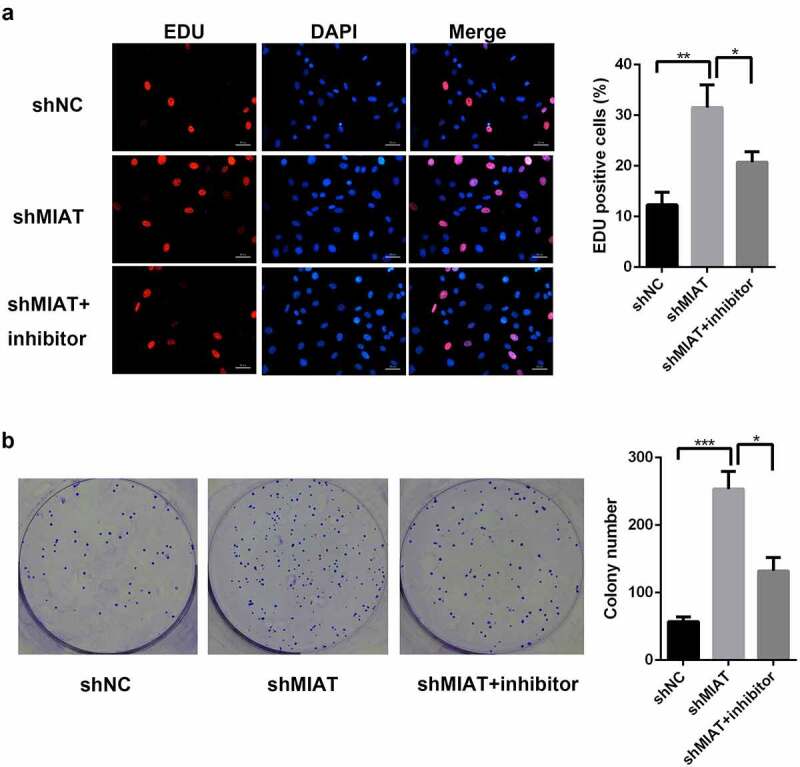
**MIAT can regulate the cell proliferation of BMSCs by targeting miR-150-5p**. EdU staining (a and b) and colony formation (c and d) showed that compared with shNC group, the proliferation of BMSCs was significantly increased in shMIAT group, which was reversed by miR-150-5p inhibitor. *p < 0.05, **p < 0.01, ***p < 0.001. Note: The petri dish is 35 mm in diameter. The scale length is 50 μm.

Based on EdU staining (Figure 5a and b) and the colony formation assay (Figure 5c and d), shMIAT infection significantly enhanced BMSC proliferation in the shMIAT group when compared with the shNC group; the miR-150-5p inhibitor partially inhibited the proliferative behaviors of shMIAT.

### 3.6 MIAT inhibits the apoptosis of BMSCs by targeting miR-150-5p

**Figure 6.**
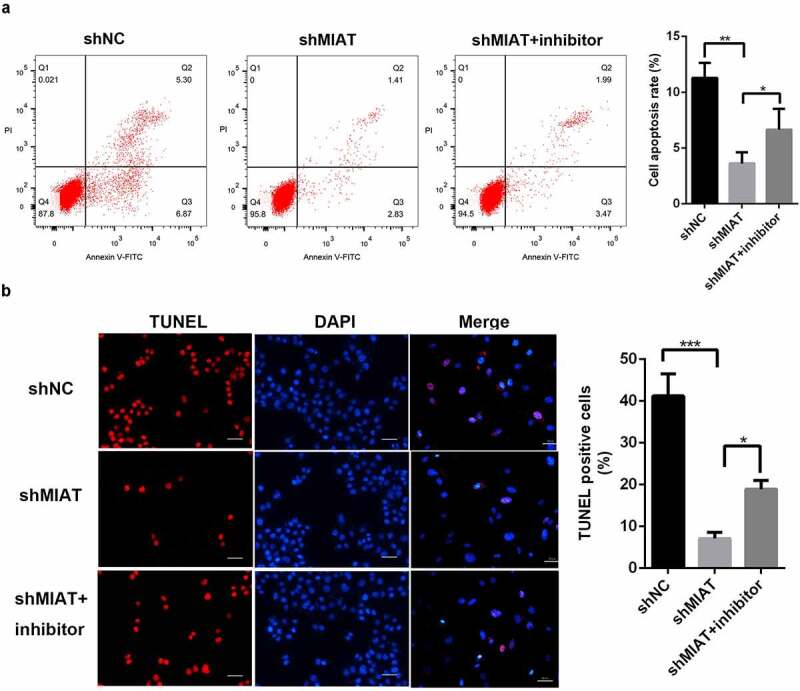
**MIAT can regulate the apoptosis of BMSCs by targeting miR-150-5p**. TUNEL staining (a and b) and flow cytometry (c and d) showed that compared with shNC group, the cell apoptosis of BMSCs was significantly decreased in shMIAT group, which was reversed by miR-150-5p inhibitor. *p < 0.05, **p < 0.01, ***p < 0.001. Note: The scale length is 50 μm.

TUNEL staining (Figure 6a and b) and flow cytometry (Figure 6c and d) showed that apoptosis of BMSCs was markedly decreased in the shMIAT group. Notably, the miR-150-5p inhibitor partially blocked the anti-apoptotic effects of shMIAT.

### 3.7 MIAT may regulate osteogenic differentiation of BMSCs by targeting miR-150-5p

**Figure 7.**
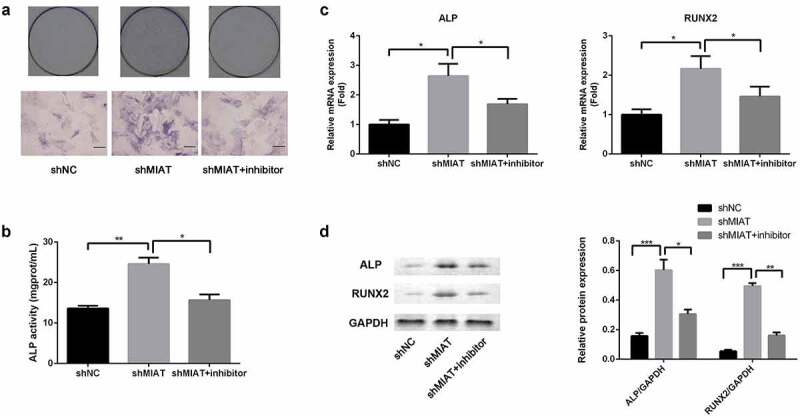
**MIAT can regulate the osteogenic differentiation of BMSCs by targeting miR-150-5p**. Compared with shNC group, the ALP activity was significantly increased in shMIAT group, which was reversed by miR-150-5p inhibitor (a and b). Compared to shNC, shMIAT significantly increased the mRNA expression of RUNX2 and ALP (c), which were reversed by miR-150-5p inhibitor. MIAT knockdown significantly promoted the protein expression of RUNX2 and ALP by targeting miR-150-5p (d). *p < 0.05, **p < 0.01, ***p < 0.001.

**Note**: The petri dish is 35 mm in diameter. The scale length is 200 μm.

ALP staining and ALP activity assay were conducted to evaluate osteogenic differentiation of BMSCs. Based on ALP staining and ALP activity assay, ALP activity was significantly increased in the shMIAT group when compared with the shNC group. Moreover, transfection of miR-150-5p inhibitor abrogated the MIAT-induced effects on ALP activity (Figure 7a and b). Finally, the role of MIAT in the expression of osteogenic markers was evaluated by RT-PCR and Western blotting. Compared with shNC, shMIAT significantly increased the BMSC expression of osteogenic markers, RUNX2 and ALP (mRNA expression (Figure 7c) and protein expression (Figure 7d) at day 14, and the osteogenic effects of MIAT were partially abrogated by transfection with miR-150-5p.

## 4. Discussion

In the present study, we reported that lncRNA MIAT was significantly increased in patients with OP and could affect the growth, cell death, and osteogenic differentiation of BMSCs by regulating miR-150-5p expression.

It is well-established that BMSCs differentiate into osteoblasts [29]. Therefore, the induction of BMSC proliferation and osteogenic differentiation is crucial for preventing and treating OP [20]. Notably, lncRNA MIAT is a novel regulator of multiple human disorders [30–32]. For example, MIAT is involved in myocardial infarction [33] and microvascular dysfunction [34]. In addition, MIAT can impact the osteogenic differentiation of human ASCs [12]. The silencing of MIAT reportedly promotes the osteogenesis of MSCs [14]. Moreover, MIAT sponges miR-150-5p in human lens epithelial cells [18]. Meanwhile, miR-150 induces osteoblast differentiation [19] and suppresses osteoclast differentiation [20]. However, it remains unclear whether MIAT regulates miR-150 in BMSCs to participate in the pathogenesis of OP.

Herein, we observed that MIAT targeted miR-150-5p, which is in line with previous study [18]. In addition, the expression of MIAT was increased, while that of miR-150-5p was decreased in patients with OP. Osteogenic differentiation indicators were also increased in serum of patients with OP. Furthermore, the expression of MIAT was decreased, while miR-150-5p was increased in BMSCs during osteogenic differentiation in a time-dependent manner. Thence, we speculated that MIAT and miR-150-5p may participate in osteogenic differentiation of OP.

Runx2 is an osteogenic marker related to osteoblast activation during osteogenesis [35], and ALP is a marker for early osteogenic differentiation of BMSCs and plays a role in the *in vitro* calcification of BMSCs [36]. In present study, MIAT was decreased and miR-150-5p was increased time-dependently during the osteogenic differentiation of BMSCs. Furthermore, MIAT knockdown significantly promoted osteogenic differentiation of BMSCs. Interestingly, co-transfection of miR-150-5p abrogated the osteogenic ability of MIAT shRNA. Therefore, our findings revealed that MIAT might affect the osteogenic differentiation of BMSCs by targeting miR-150-5p.

Abnormal bone reconstruction [37,38] is another important factor in the development of OP. In the present study, MIAT knockdown significantly increased BMSC growth and decreased apoptosis, while miR-150-5p partially blocked the proliferative and anti-apoptotic effects of MIAT shRNA. Collectively, our results indicate that MIAT can affect the growth and apoptosis of BMSCs by targeting miR-150-5p.

There are some limitations in this study. Firstly, the clinical samples were not ethnically diverse, and only Han was included. In future experiments, the sample size should be further expanded and the number of ethnic groups should be increased to verify the results of this study. Secondly, whether MIAT could regulate OP process *in vivo* by binding with miR-150 should be further investigated. Finally, the downstream gene of miR-150 that affect osteogenic differentiation of BMSCs should be studied.

## Conclusions

In summary, MIAT was downregulated while miR-150-5p was upregulated in a time-dependent manner during the osteogenic differentiation of BMSCs. Furthermore, MIAT knockdown significantly increased ALP activity, proliferation, osteogenic differentiation, and osteogenesis and inhibited apoptosis in BMSCs by targeting miR-150-5p. These results suggest the potential role of MIAT in the treatment of OP.

## Data Availability

All data generated or analyzed during this study are available from the corresponding author upon reasonable request.

## Research Highlights

1. Compared with controls, the expression of lncRNA MIAT is significantly increased in patients with osteoporosis, whereas that of miR-150-5p is markedly decreased.
2. LncRNA MIAT can bind with miR-150-5p, and the expression levels of each other exhibited a strong negative correlation.
3. Downregulation of lncRNA MIAT dramatically promotes osteogenic differentiation, increases proliferation, and inhibits apoptosis in BMSCs by sponging miR-150-5p

## References

[1] Cosman F, de Beur SJ, LeBoff MS, et al. Clinician’s guide to prevention and treatment of osteoporosis. Osteoporos Int. 2014;25(10):2359–2381. 25182228 10.1007/s00198-014-2794-2 PMC4176573

[2] Ge DW, Wang WW, Chen HT, et al. Functions of microRNAs in osteoporosis. Eur Rev Med Pharmacol Sci. 2017;21:4784–4789. 29164586

[3] Mundy GR, Elefteriou F. Boning up on ephrin signaling. Cell. 2006;126:441–443. 16901775 10.1016/j.cell.2006.07.015

[4] Pagani F, Francucci CM, Moro L. Markers of bone turnover: biochemical and clinical perspectives. J Endocrinol Invest. 2005;28(10 Suppl):S8–S13. 16550716

[5] Zhang RF, Wang Q, Zhang AA, et al. Low-level laser irradiation promotes the differentiation of bone marrow stromal cells into osteoblasts through the APN/Wnt/β‑catenin pathway. Eur Rev Med Pharmacol Sci. 2018;22:2860–2868. 29771444 10.26355/eurrev_201805_14988

[6] Wang R, Xu B, Xu HG. Up-Regulation of TGF‑β promotes Tendon-to-Bone healing after anterior cruciate ligament reconstruction using Bone Marrow-D erived mesenchymal stem cells through the TGF-β/MAPK signaling pathway in a New Zealand White Rabbit model. Cell Physiol Biochem. 2017;41:213–226. 28214835 10.1159/000456046

[7] Fan JZ, Yang L, Meng GL, et al. Estrogen improves the proliferation and differentiation of hBMSCs derived from postmenopausal osteoporosis through notch signaling pathway. Mol Cell Biochem. 2014;392:85–93. 24752351 10.1007/s11010-014-2021-7 PMC4053611

[8] Ishii N, Ozaki K, Sato H, et al. Identification of a novel non-coding RNA, MIAT, that confers risk of myocardial infarction. J Hum Genet. 2006;51:1087–1099. 17066261 10.1007/s10038-006-0070-9

[9] Yan BA, Yao J, Liu JY, et al. lncRNA-miat regulates microvascular dysfunction by functioning as a competing endogenous RNA. Circ Res. 2015;116(7):1143–1156. 25587098 10.1161/CIRCRESAHA.116.305510

[10] Rao SQ, Hui HL, Ye N, et al. Genetic variants in long non-coding RNA MIAT contribute to risk of paranoid schizophrenia in a Chinese Han population. Schizophr Res. 2015;166:125–130. 26004688 10.1016/j.schres.2015.04.032

[11] Lai IL, Yang CA, Lin PC, et al. Long noncoding RNA MIAT promotes non-small cell lung cancer proliferation and metastasis through MMP9 activation. Oncotarget. 2017;8:98148–98162. 29228680 10.18632/oncotarget.21465 PMC5716720

[12] Jin C, Zheng Y, Huang Y, et al. Long non-coding RNA MIAT knockdown promotes osteogenic differentiation of human adipose-derived stem cells. Cell Biol Int. 2017;41(1):33–41. 27797128 10.1002/cbin.10697

[13] Ishii N, Ozaki K, Sato H, et al. Identification of a novel non-coding RNA, MIAT, that confers risk of myocardial infarction. J Hum Genet. 2006;51(12):1087–1099. 17066261 10.1007/s10038-006-0070-9

[14] Fang B, Li Y, Chen C, et al. Huo Xue Tong Luo capsule ameliorates osteonecrosis of femoral head through inhibiting lncRNA-Miat. J Ethnopharmacol. 2019;238:111862. 30970282 10.1016/j.jep.2019.111862

[15] Jia J, Tian Q, Ling S, et al. MiR-145 suppresses osteogenic differentiation by targeting Sp7. FEBS Lett. 2013;587:3027–3031. 23886710 10.1016/j.febslet.2013.07.030

[16] Chen L, Holmstrom K, Qiu W, et al. MicroRNA-34a inhibits osteoblast differentiation and in vivo bone formation of human stromal stem cells. Stem Cells. 2014;32:902–912. 24307639 10.1002/stem.1615

[17] Wang H, Ding XG, Yang JJ, et al. LncRNA MIAT facilitated BM-MSCs differentiation into endothelial cells and restored erectile dysfunction via targeting miR-200a in a rat model of erectile dysfunction. Eur J Cell Biol. 2018;97:180–189. 29486902 10.1016/j.ejcb.2018.02.001

[18] Shen Y, Dong LF, Zhou RM, et al. Role of long noncoding RNA MIAT in proliferation, apoptosis and migration of lens epithelial cells: a clinical and in vitro study. J Cell Mol Med. 2016;20(3):537–548. 26818536 10.1111/jcmm.12755 PMC4759467

[19] Dong CL, Liu HZ, Zhang ZC, et al. The influence of MicroRNA-150 in Osteoblast Matrix Mineralization. J Cell Biochem. 2015;116(12):2970–2979. 26212040 10.1002/jcb.25245

[20] Choi SW, Lee SU, Kim EH, et al. Osteoporotic bone of miR-150-deficient mice: possibly due to low serum OPG-mediated osteoclast activation. Bone Rep. 2015;3:5–10. 28377961 10.1016/j.bonr.2015.06.003 PMC5365209

[21] Mao Y, Yan R, Li A, et al. Lentiviral Vectors Mediate Long-Term and High Efficiency Transgene Expression in HEK 293T cells. Int J Med Sci. 2015;12(5):407–415. 26005375 10.7150/ijms.11270 PMC4441065

[22] Zhang L, Tang Y, Zhu X, et al. Overexpression of MiR-335-5p Promotes Bone Formation and Regeneration in Mice. J Bone Miner Res. 2017;32(12):2466–2475. 28846804 10.1002/jbmr.3230 PMC5732062

[23] Chen L, Zhang D, Yu L, et al. Targeting MIAT reduces apoptosis of cardiomyocytes after ischemia/reperfusion injury. Bioengineered. 2019;10(1):121–132. 30971184 10.1080/21655979.2019.1605812 PMC6527071

[24] Chen J, Chen T, Zhu Y, et al. circPTN sponges miR-145-5p/miR-330-5p to promote proliferation and stemness in glioma. J Exp Clin Cancer Res. 2019;38(1):398. 31511040 10.1186/s13046-019-1376-8 PMC6737709

[25] Zhang X, Wang S, Wang H, et al. Circular RNA circNRIP1 acts as a microRNA-149-5p sponge to promote gastric cancer progression via the AKT1/mTOR pathway. Mol Cancer. 2019;18(1):20. 30717751 10.1186/s12943-018-0935-5 PMC6360801

[26] Zhang SF, Zhang K, Cheng HM, et al. Comparative transcriptomics reveals colony formation mechanism of a harmful algal bloom species Phaeocystis globosa. Sci Total Environ. 2020;719:137454. 32114233 10.1016/j.scitotenv.2020.137454

[27] Nakamura T, Nakamura-Takahashi A, Kasahara M, et al. Tissue-nonspecific alkaline phosphatase promotes the osteogenic differentiation of osteoprogenitor cells. Biochem Biophys Res Commun. 2020;524(3):702–709. 32035618 10.1016/j.bbrc.2020.01.136

[28] Liang J, Chen C, Liu H, et al. Gossypol Promotes Bone Formation in Ovariectomy-Induced Osteoporosis through Regulating Cell Apoptosis. Biomed Res Int. 2018;2018:3635485. 30643801 10.1155/2018/3635485 PMC6311247

[29] Xu R, Shi G, Xu L, et al. Simvastatin improves oral implant osseointegration via enhanced autophagy and osteogenesis of BMSCs and inhibited osteoclast activity. J Tissue Eng Regen Med. 2018;12:1209–1219. 29498229 10.1002/term.2652

[30] Wang R, Xu B, Xu HG. Up-Regulation of TGF-β promotes Tendon-to-Bone healing after anterior cruciate ligament reconstruction using Bone Marrow-D erived mesenchymal stem cells through the TGF-β/MAPK signaling pathway in a New Zealand White Rabbit model. Cell Physiol Biochem. 2017;41:213‑226. 10.1159/000456046 28214835

[31] Wapinski O, Chang HY. Long noncoding RNAs and human disease. Trends Cell Biol. 2011;21:354–361. 21550244 10.1016/j.tcb.2011.04.001

[32] Esteller M. Non-coding RNAs in human disease. Nat Rev Genet. 2011;12:861–874. 22094949 10.1038/nrg3074

[33] Liao J, He Q, Li M, et al. LncRNA MIAT: myocardial infarction associated and more. Gene. 2016;578(2):158–161. 26707210 10.1016/j.gene.2015.12.032

[34] Yan B, Yao J, Liu JY, et al. LncRNA-MIAT regulates microvascular dysfunction by functioning as a competing endogenous RNA. Circ Res. 2015;116(7):1143–1156. 25587098 10.1161/CIRCRESAHA.116.305510

[35] Mundy GR, Elefteriou F. Boning up on ephrin signaling. Cell. 2006;126:441–443. 16901775 10.1016/j.cell.2006.07.015

[36] Bai Y, Yin G, Huang Z, et al. Localized delivery of growth factors for angiogenesis and bone formation in tissue engineering. Int Immunopharmacol. 2013;16:214–223. 23587487 10.1016/j.intimp.2013.04.001

[37] Qiu J, Huang G, Na N, et al. MicroRNA‑ 214‑5p/TGF‑β/Smad2 signaling alters adipogenic differentiation of bone marrow stem cells in postmenopausal osteoporosis. Mol Med Rep. 2018;17:6301‑6310. 10.3892/mmr.2018.8713 PMC5928609 29532880

[38] Abdi J, Mutis T, Garssen J, et al. Toll‑like receptor (TLR)‑1/2 triggering of multiple myeloma cells modulates their adhesion to bone marrow stromal cells and enhances bortezomib‑induced apoptosis. PLoS One. 2014;9:e96608. 24794258 10.1371/journal.pone.0096608 PMC4008602

